# Enhancing groundwater vulnerability assessment for improved environmental management: addressing a critical environmental concern

**DOI:** 10.1007/s11356-024-32305-1

**Published:** 2024-02-15

**Authors:** Yasir Abduljaleel, Mustapha Amiri, Ehab Mohammad Amen, Ali Salem, Zana Fattah Ali, Ahmed Awd, Dénes Lóczy, Mohamed Ghzal

**Affiliations:** 1 Department of Civil and Environmental Engineering, Washington State University, Richland, WA 99354 USA; 2 Geomatics and Soil Management Laboratory, Faculty of Arts and Humanities, Université Mohammed Premier Oujda, 60000 Oujda, Morocco; 3 Natural Resources Research Center (NRRC), Tikrit University, Tikrit, 34001 Iraq; 4 Departamento de Geodinámica, Universidad de Granada, Granada, 18071 Spain; 5 Department of Applied Geology, Collage of Science, Tikrit University, Tikrit, 34001 Iraq; 6 Civil Engineering Department, Faculty of Engineering, Minia University, Minia, 61111 Egypt; 7 Structural Diagnostics and Analysis Research Group, Faculty of Engineering and Information Technology, University of Pécs, Boszorkány ut 2, 7624 Pecs, Hungary; 8 Department of Geography, Faculty of Education, Koya University, Koysinjaq, 46011 Iraq; 9 Doctoral School of Earth Sciences, University of Pécs, Ifjúság útja 6, 7624 Pécs, Hungary; 10 Department of Food, Agriculture and Biological Engineering (FABE), The Ohio State University, Columbus, 43210 USA; 11 Egyptian Ministry of Water Resources and Irrigation (MWRI), Giza, 11925 Egypt; 12 Institute of Geography and Earth Sciences, Faculty of Sciences, University of Pécs, Ifjúság útja 6, 7624 Pécs, Hungary

**Keywords:** Groundwater vulnerability, Environmental management, El Orjane aquifer, DRASTIC, SINTACS, SI

## Abstract

Groundwater serves as a primary water source for various purposes. Therefore, aquifer pollution poses a critical threat to human health and the environment. Identifying the aquifer’s highly vulnerable areas to pollution is necessary to implement appropriate remedial measures, thus ensuring groundwater sustainability. This paper aims to enhance groundwater vulnerability assessment (GWVA) to manage aquifer quality effectively. The study focuses on the El Orjane Aquifer in the Moulouya basin, Morocco, which is facing significant degradation due to olive mill wastewater. Groundwater vulnerability maps (GVMs) were generated using the DRASTIC, Pesticide DRASTIC, SINTACS, and SI methods. To assess the effectiveness of the proposed improvements, 24 piezometers were installed to measure nitrate concentrations, a common indicator of groundwater contamination. This study aimed to enhance GWVA by incorporating new layers, such as land use, and adjusting parameter rates based on a comprehensive sensitivity analysis. The results demonstrate a significant increase in Pearson correlation values (PCV) between the produced GVMs and measured nitrate concentrations. For instance, the PCV for the DRASTIC method improved from 0.42 to 0.75 after adding the land use layer and adjusting parameter rates using the Wilcoxon method. These findings offer valuable insights for accurately assessing groundwater vulnerability in areas with similar hazards and hydrological conditions, particularly in semi-arid and arid regions. They contribute to improving groundwater and environmental management practices, ensuring the long-term sustainability of aquifers.

**Supplementary Information:**

The online version contains supplementary material available at 10.1007/s11356-024-32305-1.

## Introduction

Groundwater is considered one of the world’s finest potable natural resources and is often the most critical water source when planning water supply systems, especially in arid and semi-arid regions (Ruidas et al. 2023). According to Shen et al. (2008), over one and a half billion people depend directly and indirectly on groundwater. However, according to UNESCO’s 2015 estimation, at least 50% of the global population heavily relies on groundwater for drinking purposes due to its abundance and lower vulnerability to pollution compared to surface waters (Ruidas et al. 2023; Zamani et al. 2022). However, groundwater is deteriorating globally at an alarming rate. Unfortunately, shallow aquifer groundwater has been severely affected in recent decades due to both geogenic and anthropogenic reasons (Ruidas et al. 2024). Groundwater vulnerability is a term used to describe the sensitivity of a groundwater system to degradation by pollutants originating from human activities (Hirata and Bertolo 2009). The National Research Council (Council 1993) provides another definition of groundwater vulnerability as the relative ease with which a contaminant (e.g., a pesticide) applied on or near the land surface can migrate to the aquifer of interest under a given set of agronomic management practices, pesticide characteristics, and hydrogeological sensitivity conditions.

Various types of groundwater vulnerability have been identified in the scientific literature. Intrinsic vulnerability refers to the inherent geological, hydrological, hydrogeological, and hydrogeochemical characteristics of an area (Abu-Bakr 2020; Taghavi et al. 2022). In recent years, the global population has increased, leading to a surge in human activities in various sectors, including agriculture and industry (Salem et al. 2023). This has resulted in the production of hazardous chemical materials that can infiltrate porous aquifers, causing a significant deterioration in groundwater quality (Chen et al. 2016). In addition, poor management practices in less developed regions, especially rural areas, lead to bacterial and nitrate (NO_3_) contamination of groundwater as a result of improper disposal of human and animal waste (Pang et al. 2013). These challenges endanger the sustainability of groundwater, exacerbating water scarcity issues faced by billions of people worldwide who lack access to surface water (Liu et al. 2017; Mancosu et al. 2015). Preserving groundwater quantity and quality is crucial for meeting diverse water needs such as drinking water supply, agriculture, and industry, especially in arid and semi-arid regions.

The Moulouya basin in Morocco is an example of an environment prone to severe groundwater degradation (Amiri et al. 2021). The arid climate in the Middle Moulouya region is characterized by low precipitation and high evaporation. This has resulted in surface water being insufficient to meet the demands of various sectors (Tekken and Kropp 2012; Salem et al. 2021). Therefore, the El Orjane aquifer plays a crucial role in providing groundwater to alleviate surface water shortages (Schyns 2013). However, the overexploitation of groundwater and the absence of an effective management system pose significant pressures on the aquifer’s sustainability.

Olive oil production poses a significant threat to groundwater sustainability in the study area due to the generation of olive mill wastewater (OMW) during the initial step of crushing olives. This activity is a key economic driver for both farmers and the region, with approximately 5492 t of olives being crushed annually between November and February. The washing process of olives after harvesting poses a risk due to the haphazard disposal of untreated wastewater. OMW is widely recognized as the most polluting effluent generated by the olive industry (Barbera et al. 2013; Chatzistathis and Koutsos 2017). It contains polyphenols with concentrations reaching up to 18 g/L and has pH levels ranging from 3 to 6. Additionally, OMW exhibits high chemical oxygen demand (COD) values that can exceed 220 g/L (Al-Khatibet al. 2009). The indiscriminate disposal of raw OMW in the study area poses significant environmental risks to watercourses, groundwater, soil, and public sewerage systems due to the large volume of OMW produced (reaching 1.8 m^3^/t of olives).

Groundwater monitoring and sampling can reveal aquifer vulnerability, but it is a complex and complicated process. Numerous models have been developed to facilitate aquifer vulnerability assessment (Gogu and Dassargues 2000; Machiwal et al. 2018; Maria 2018). These approaches can be categorized as follows: (i) GIS-based qualitative methods, (ii) statistical methods, (iii) process-based numerical models, and (iv) process-based models. Among these, GIS-based qualitative methods have been found to be effective in determining groundwater vulnerability. They are relatively easy to apply and are not limited by computational complexity or data scarcity compared to other methods. Furthermore, researchers have utilized GIS-based qualitative methods as a foundation for developing machine-learning models to assess groundwater vulnerability (Das and Pal 2020, 2019). For instance, Elzain et al. (2022) employed three machine learning models, radial basis neural networks (RBNN), support vector regression (SVR), and ensemble random forest regression (RFR) all of which are based on the DRASTIC-L model, to evaluate the groundwater vulnerability in the Miryang area of Korea. Bordbar et al. (2022) conducted a study that integrated an adaptive neuro-fuzzy inference system (ANFIS), support vector machine (SVM), and artificial neural network (ANN) to design an integrated supervised committee machine artificial intelligence (SCMAI) for spatially predicting groundwater vulnerability in Gharesoo-Gorgan Rood coastal aquifer located in the northern part of Iran. Band et al. (2020) assessed the suitability of the fuzzy-AHP technique for evaluating groundwater recharge potential zones in the groundwater-stressed Goghat-II block, West Bengal, India.

The DRASTIC model, developed by the United States Environmental Protection Agency (USEPA), is a commonly used GIS-based qualitative method for assessing groundwater vulnerability. The DRASTIC model considers several parameters, including groundwater table depth (*D*), net recharge (*R*), aquifer medium (*A*), soil media (*S*), topography (*T*), impact of the vadose zone (*I*), and hydraulic conductivity variations (*C*) (Patel et al. 2022). The model’s name is derived from the abbreviations of these parameters. The Pesticide DRASTIC model also considers the same geological, hydrological, and climate parameters as the original DRASTIC model but assigns different weights to these parameters. Similarly, the SINTACS model uses the same seven parameters as the DRASTIC model but assigns different weights to them. The SI method is a modified version of DRASTIC that considers three parameters: vadose zone, hydraulic conductivity, and soil media are omitted, and a land use layer is added.

Researchers’ efforts continued to improve the GIS-based groundwater vulnerability assessment for better groundwater management (Albuquerque et al. 2021; Dhaoui et al. 2022). For instance, Abdullah et al. (2018) have tested different approaches of weighting techniques for the DRASTIC index. That is besides introducing different DRASTIC calibration techniques and other approaches such as the modified SINTACS method and the susceptibility index (SI) (Voudouris et al. 2018).

The first objective of this study is to employ GIS-based qualitative methods to create a groundwater vulnerability map of the study area using the following methods:

(i) DRASTIC model
(ii) Pesticide DRASTIC model
(iii) SINTACS model
(iv) Susceptibility index (SI) model

Then and given that land use (LU) is not considered in the three methods (DRASTIC, Pesticide DRASTIC, and SINTACS), the second objective of this study is to enhance the sensitivity of these methods to field conditions by incorporating a LU layer. Subsequently, the modified methods (DRASTIC-LU, Pesticide DRASTIC-LU, and SINTACS-LU) will be evaluated by comparing the resulting groundwater vulnerability maps (GVMs( with measured nitrate concentrations in groundwater samples collected from 24 locations in the study area. The decision to validate the GVMs using nitrate concentrations is based on the intensive agricultural activities in the region. Excessive and unregulated fertilizer use in the area can potentially lead to significant nitrate pollution in groundwater. This is supported by previous studies (Sebou 2011; Schyns 2013).

Finally, this study uses the analytical hierarchy process (AHP) and Wilcoxon techniques to adjust the weight and rating coefficients of the layers included in the groundwater vulnerability models to enhance their performance. These adjustments are based on a thorough sensitivity analysis to evaluate the effects of each layer.

## Study area and methodology

### Study area

**Fig. 1:**
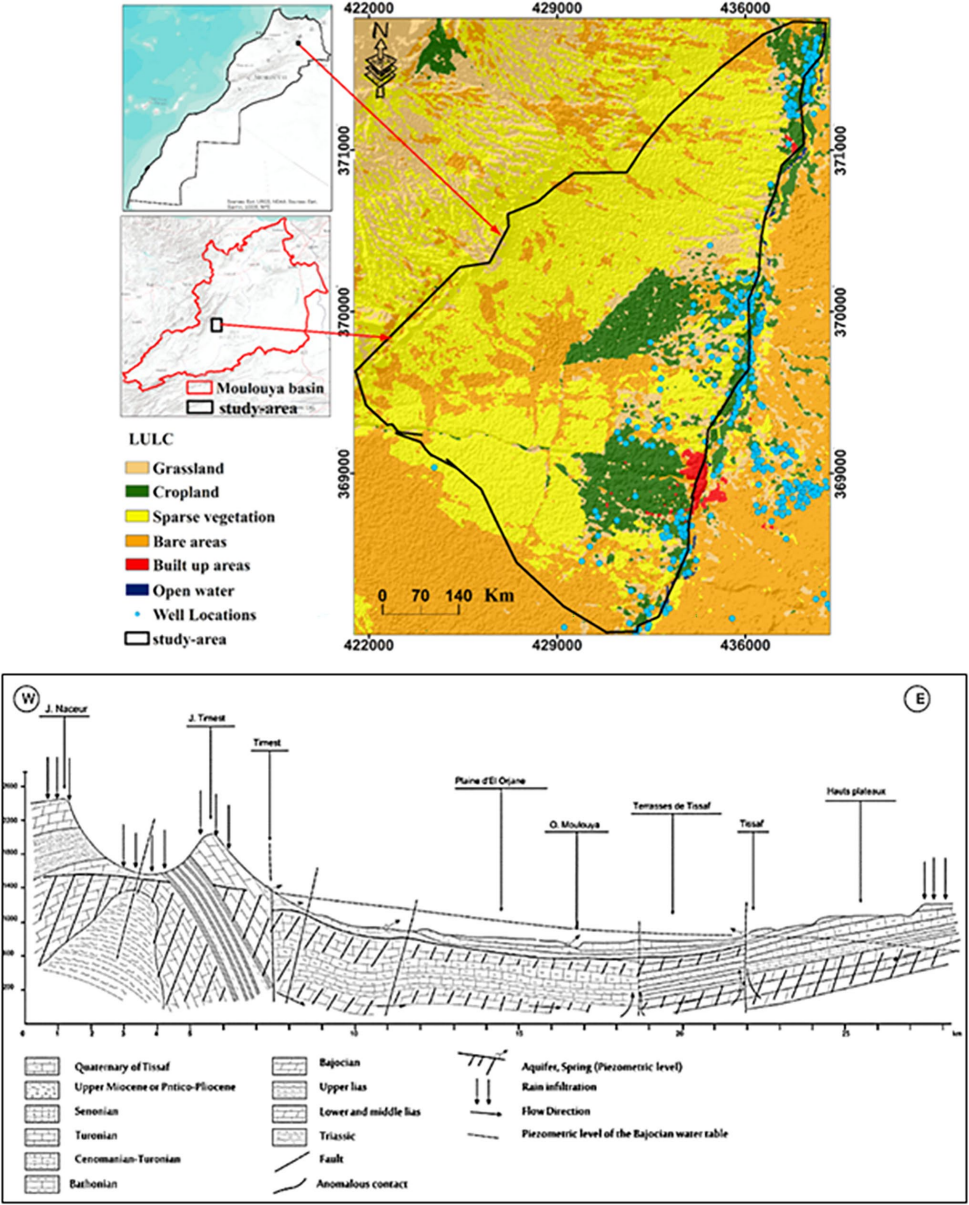
Study area; location and lithology. Modified after Combe (1975)

The research focuses on the El Orjane aquifer, located on the left bank of the Moulouya basin in Morocco (Fig. 1). The aquifer consists of Miocene conglomerate and sandstone formations. The study area covers approximately 184.95 km^2^, ranging from coordinates 644737 E to 6,611917 E and 323353 N to 300535 N. The region has an arid climate, with average annual precipitation, temperature, and evapotranspiration values of 198 mm, 18 °C, and 166 mm, respectively.

Groundwater is a vital water source for agricultural, industrial, and domestic activities in the study area. Olive cultivation is the dominant agricultural activity, with olive oil production being a significant source of income for farmers and the country. The study area has three types of olive oil production units: traditional, semi-modern, and modern, with 207, 27, and 18 units, respectively. The annual production of olive oil from these units exceeds 17,000 t. The substantial amount of olive oil produced and the subsequent waste, both liquid and solid, infiltrate into the soil layers, threatening the groundwater quality in the study area.

### Methodology

**Fig. 2:**
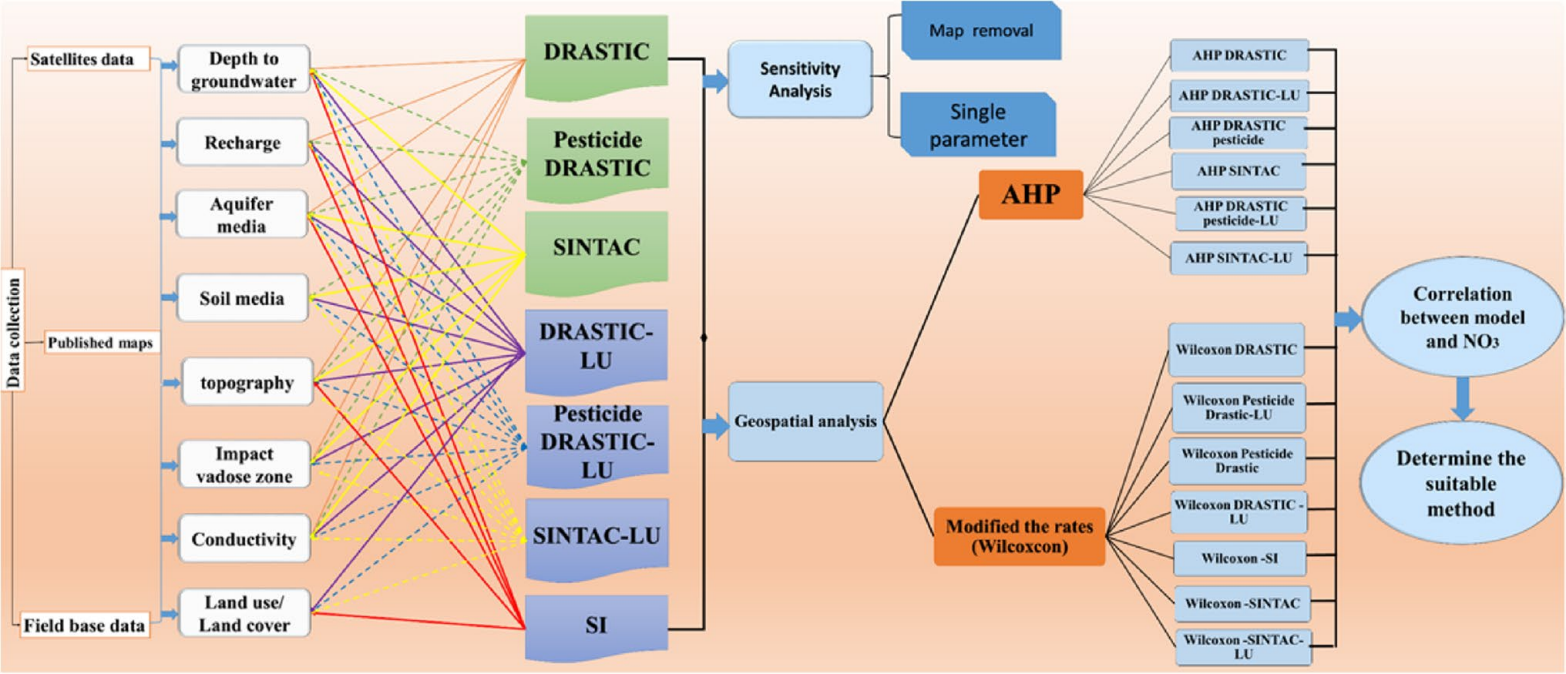
The proposed methodology

To accomplish the objective of this study, we followed the methodology presented in Fig. 2. Initially, we generated GVMs from the following models:

- DRASTIC
- Pesticide DRASTIC
- SINTACS

As reported by many researchers, including Saha and Alam (2014) and Hamza et al. (2015), the DRASTIC model is a numerical model developed by Aller et al. (1987) that assesses the degree of groundwater vulnerability to pollution at various scales, including local, regional, and global (Nagar and Mirza, 2002). The model assigns a weight from 1 to 5 for each factor, indicating the potential vulnerability of groundwater. A low-weight factor suggests a low possibility of groundwater vulnerability, while a high-weight factor indicates the opposite. Moreover, each factor is further divided into sub-layers, and each sub-layer is assigned a weight from 1 to 10, representing its relative contribution to groundwater contamination. By employing Eq. (1), the DRASTIC model calculates the groundwater vulnerability index (VI):

1 $$\mathrm{DRASTIC \;VI}=Dr \times Dm+Rr \times Rm+Ar \times Af+Sr \times Sm+Tr \times Tm+Ir \times Im+Cr \times Cm$$

where

*D*, *R*, *A*,* S*, *T*,* I*, and *C* refer to the seven factors that are considered in DRASTIC.

*r*: refers to the rate of factors.
*m*: refers to the weight of factors. Pesticide

DRASTIC considers the same DRASTIC factors, but with assigning different weights for these parameters.

Table 1 shows the weight of these factors for both DRASTIC and Pesticide DRASTIC.

**Table 1:** Weight of factors for groundwater VI determination in DRASTIC, Pesticide DRASTIC, and SINTACS models

Factor	Weight		
	DRASTIC	Pesticide DRASTIC	SINTACS
Depth to groundwater	5	5	5
Net recharge	4	4	4
Aquifer media	3	3	3
Soil media	2	5	4
Topography	1	3	2
Impact of vadose zone	5	4	5
Hydraulic conductivity	3	2	3

The SINTACS model was developed in Italy by Civita (Aller et al. 1987) and further enhanced by Civita et al. (Civita et al. 1999). SINTACS is a modified version of the DRASTIC model, specifically tailored to adapt to the unique conditions found in Mediterranean regions. It has been applied by many researchers, including Kumar et al. (2013), Ewusi et al. (2017), and Awawdeh et al. (2020). The SINTACS method distinguishes itself by varying the weights assigned to its seven parameters in different scenarios. Equation (2) is used to calculate the groundwater VI in SINTACS.

2 $$\mathrm{SINTACS\; VI}= {\sum }_{i=1}^{7}Pi*Wi$$

where

*P*: refers to the rating of each of the seven parameters that are considered in the SINTACS method.
*W*: is the relative weight of these parameters (Table 1).

Due to the significant impact of land use patterns on groundwater vulnerability, the performance of the aforementioned methods was enhanced by incorporating a LU layer following the guidelines outlined by Secunda et al. (Secunda et al. 1998). Table 2 displays the parameter weights for the DRASTIC, Pesticide DRASTIC, and SINTACS models after the inclusion of the LU layer.

**Table 2:** Weights of factors for groundwater VI determination in DRASTIC-LU, Pesticide DRASTIC-LU, SINTACS-LU, and SI models

Factor	Weight			
	DRASTIC-LU	Pesticide DRASTIC-LU	SINTACS-LU	SI
Depth to groundwater	5	5	5	0.18
Net recharge	4	4	4	0.21
Aquifer media	3	3	3	0.25
Soil media	2	5	4	–-
Topography	1	3	2	0.12
Impact of vadose zone	5	4	5	–-
Hydraulic conductivity	3	2	3	–-
LU	5	5	5	0.22

The present research applied the SI method to produce GVMs for the study area. This method incorporates land use patterns into the assessment of groundwater vulnerability. The SI method, developed in Portugal by Ribeiro (Ribeiro 2000), is derived from the DRASTIC model. In the SI method, three out of the seven parameters considered in DRASTIC are excluded, namely, the vadose zone, hydraulic conductivity, and soil media. Instead, a land use input layer is included. Thus, the SI for groundwater vulnerability is calculated using five parameters as shown in Eq. (3).

3 $${\text{SI}}=0.186 \times D+0.212 \times R+00.259 \times A+0.121 \times T+0.222 \times {\text{LU}}$$

where

*D*, *R*, *A*, *T*, and LU are the five considered parameters in the SI method.

**Fig. 3:**
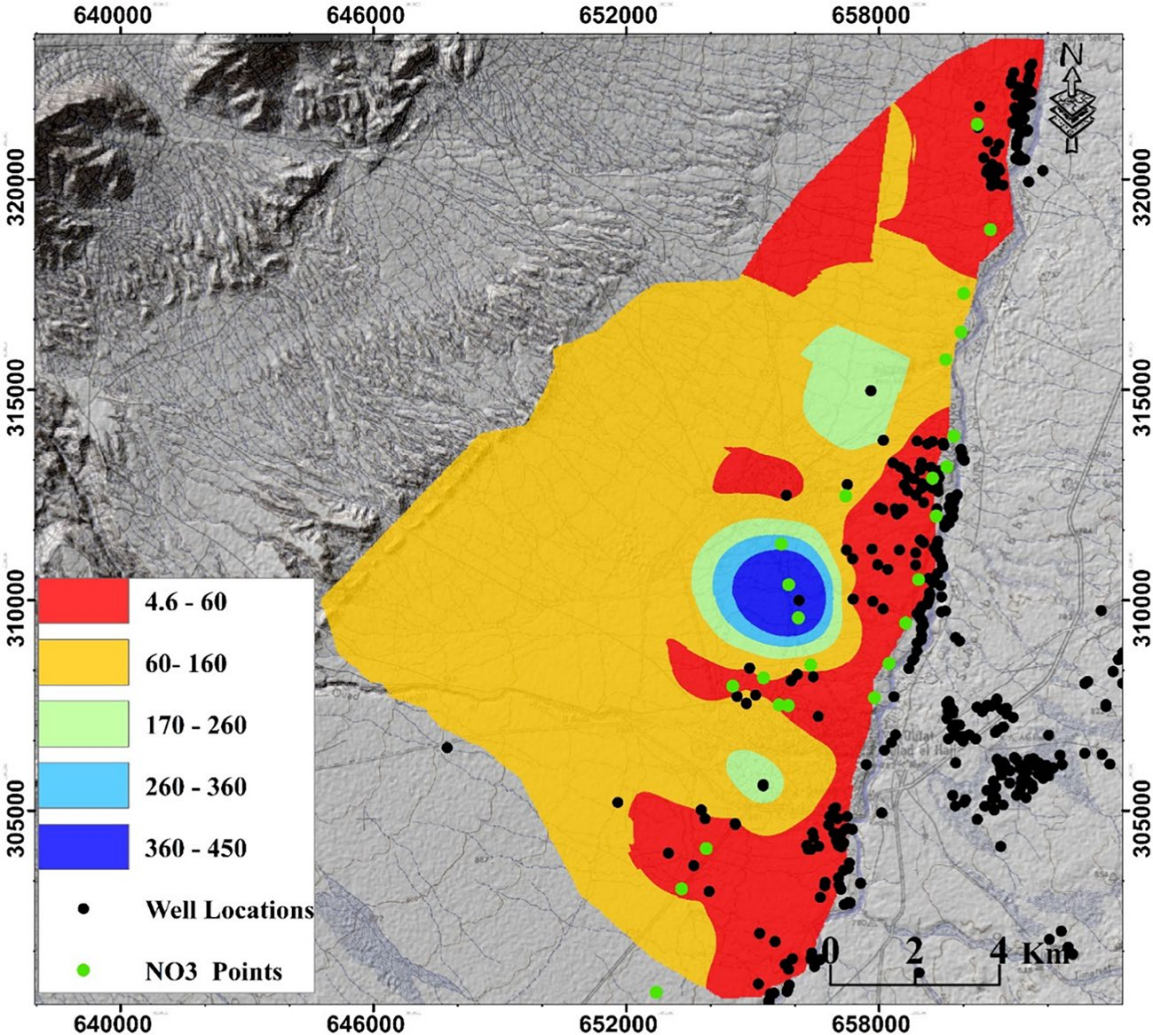
Location and details of the wells used in this study; Green dots represent the ones used for groundwater nitrate concentration measurements. Legend refers to the groundwater table depths in these wells

Nitrate concentration is commonly used as an indicator of groundwater contamination and is a critical predictor of water quality and anthropogenic pollution (Asadi et al. 2017). In this study, we evaluated the performance of the applied methods by measuring nitrate concentrations in groundwater samples collected from 24 locations within the study area. Details of these locations are shown in Fig. 3 and Table S1. Pearson’s method (Pearson 1900) was used to assess the correlation between the produced GVMs and the measured nitrate concentrations. This evaluation was carried out after each method application or improvement to determine the suitability of these methods for the specific characteristics of the study area.

For sensitivity analysis, two methods were employed: the single-parameter method and the map removal method. The single-parameter sensitivity analysis method was used to assess how changes in the model inputs affect the corresponding outputs (Huang et al. 2020). This method is important for uncertainty analysis and the development and evaluation of hydrological models (Ma et al. 2000; Wang et al. 2007). The map removal method, originally proposed by Lodwick et al. (1990), was used to examine the sensitivity of the interactions among map layers. This method evaluates the vulnerability maps’ sensitivity by selectively removing one or more layers from the applied model.

The sensitivity analysis was followed by modifying the parameter weights and improving the prediction of groundwater vulnerability. That was achieved using the two methods: (1) the AHP and (2) the Wilcoxon rank-sum nonparametric statistical test.

The AHP approach, developed by Saaty in 1980, is an effective method for analyzing complex problems with multiple interconnected goals. The AHP method consists of six steps. First, it defines the objective or phenomenon under study. Second, it determines the scale weights for each factor. Third, it calculates the geometric mean from a matrix analysis. Fourth, it ranks the criteria and sub-criteria based on the matrix calculations. Fifth, it assesses the consistency and compares biases. Finally, the weights for variable criteria and sub-criteria are evaluated based on different variables using Saaty’s 1 to 9 scale (Table S2) as described by Abrams et al. (2018). The relative weight values are assigned on a scale of 1 to 9, where 1 represents equal importance between the two variables, and 9 indicates extreme importance of one variable compared to the other (Saaty, 1980). The approach considers the consistency ratio (CR, Eq. 5) and consistency index (CI, Eq. (4)) to determine the weights of the respective variables (Saaty, 2008). CI serves as a measure of consistency in the AHP method and is derived from the following equation.

4 $$CI=\frac{\lambda max-n}{n}$$

CI in the AHP method is calculated using the equation, where CI represents the consistency index, *λ* max denotes the largest eigenvalue of the pairwise comparison matrix, and *n* indicates the number of variables or factors being considered. CR is then derived from the pairwise comparison matrices (Saaty, 1980):

5 $$CR=\frac{CI}{RI}$$

In the AHP method, CR is calculated using the ratio index (RI) and a reference table (Table S3) that provides RI values for different numbers of variables (*n*). A CR value of less than or equal to 0.01 indicates acceptable inconsistency levels.

To adjust the ratings of the parameters, the Wilcoxon rank-sum nonparametric test was employed. The method used in this study modified the ratings for each parameter by taking into account the mean concentration of nitrate in each class. The class with the highest pollutant concentration was assigned the highest rating, and the ratings for other classes were adjusted proportionally based on a linear relationship. If no nitrate concentration was observed in a class, the original DRASTIC rating was retained for that class (Neshat et al. 2014a, b). Table 3 presents the original and modified ratings of the parameters used in the applied methods, as determined by the Wilcoxon test.

**Table 3:** Original and modified rates obtained from the Wilcoxon test

Parameter	Sub-classes	Original rate	Modified rate	Mean nitrate (mg/L)
Recharge (mm/year)	0–20	1	1.0	No data
	21–40	2	2.0	No data
	41–60	3	3.7	24.5
	61–80	4	5.0	33.21
	81–130	5	5.0	No data
Slope (degrees)	0–2	5	3.7	28.21
	2_6	4	5.0	38.95
	6_12	3	1.0	7.65
	12_18	2	2.0	No data
	18_23	1	1.0	No data
Depth to groundwater (m)	4_60	5	5.0	38.12
	61_160	4	3.5	26.51
	170_260	3	3.0	No data
	270_370	2	1.6	12.056
	370_750	1	1.3	9.61
Soil media	Clay loam	4	4.0	32.83
Vadose zone	Silt	3	3.0	No data
	Alluvium	4	5.0	32.83
	Conglomerate	5	5.0	No data
Aquifer media	Conglomerates and sandstone	4	4.1	32.83
Hydraulic conductivity (m/d)	0.33_0.36	1	5.0	40.26
	0.4_0.43	2	2.1	16.83
	0.44_0.48	3	2.5	20.26
	0.49_0.53	4	4.0	No data
	0.54_0.60	5	5.0	No data
Land use	Trees cover areas	1	1.0	No data
	Shrubs cover areas/grassland	2	5.0	35.79
	Lichen mosses/sparse vegetation/bare areas	3	3.3	23.7
	Open water	4	4.0	No data
	Cropland/built-up areas	5	4.6	32.58

### Data collection and preparation

The study consists of eight major inputs that are essential to achieve its objectives. These inputs are as follows.

#### Depth to groundwater

Depth to groundwater table defined as the vertical distance from the ground surface to the water surface in an aquifer is considered the most important factor in determining groundwater vulnerability. As a general concept, contamination is less likely to affect a deeper aquifer than a shallow one (Saidi et al. 2013). In this study, we collected the groundwater depths for 300 wells from the Agency of Hydraulic Basin, Moulouya (AHBM), and interpolated them using the inverse distance weighting method.

#### Net recharge

The term “net recharge” refers to the amount of water that infiltrates and reaches the underlying aquifer, whether it is present on the land surface or in open channels. The value of “net recharge” is dynamic and influenced by various factors. It indicates the rate at which pollutants, such as those from excessive irrigation water and rainfall, are transported to aquifers (Aller et al. 1987). Higher values of “net recharge” generally indicate a greater potential for groundwater contamination, while lower values suggest a lower risk (Boufala et al. 2022). The “net recharge” in this study maps was generated using the WetSpass model, taking into account the findings reported by Amiri et al. (2022).

#### Aquifer media

The term “aquifer media” refers to the lithology or geological composition of the aquifer, which represents the material in which water is stored. This medium can consist of various materials, including unconsolidated rocks, consolidated formations, pebbles, and other geological elements. The properties of aquifer media play a crucial role in influencing the flow path and rate of water, as well as the movement of pollutants within the aquifer. According to Bera et al. (2021), lithologies with slower water are generally considered less susceptible to contamination. The El Orjane aquifer is primarily composed of Miocene conglomerates and sandstones, as indicated by the aquifer map. The geological formations present in the study area are expected to have an impact on the movement and distribution of groundwater. This is crucial for assessing the vulnerability of groundwater to pollution.

#### Soil media

The soil acts as the primary medium for water infiltration and movement into the vadose zone, eventually reaching the aquifers. Therefore, the soil media plays a critical role in determining the quantity of water and pollutants that enter the aquifer (Nahin et al. 2020). Soil media that permit swift infiltration of water from the surface to the underlying layers are deemed more vulnerable, while media that hinder such rapid infiltration are considered less vulnerable. In this study, we collected data on soil media using the FAO Digital Soil Map of the World (DSMW) (Nachtergaele et al. 2023).

#### Topography

Water present on land surfaces, which contains pollutants, typically follows two paths: (1) it may accumulate and infiltrate underground, or (2) it may flow downhill as runoff. The path it takes is primarily determined by the land topography, specifically the longitudinal slopes of the land tract (Thapa et al. 2018). Flat land areas that receive runoff from adjacent lands can pose a significant threat to the quality of the underlying aquifer. These flat lands collect a significant amount of pollutants from the surrounding areas, which are carried by the runoff and subsequently infiltrate underground, settling in the aquifer layers. The topography data for the study area were generated using the Shuttle Radar Topography Mission (SRTM) with a resolution of 30 m × 30 m.

#### Vadose zone

The term “vadose zone” refers to the unsaturated zone that extends from the soil surface to the groundwater table. The soil media in the vadose zone plays a crucial role in reducing pollution through processes such as biodegradation, mechanical filtration, sorption, volatilization, and dispersion. As a result, the vadose zone significantly affects groundwater vulnerability. Aquifers located beneath soil media allowing easy filtration and downward movement of pollutants are considered highly vulnerable to contamination. This study utilized the lithology map provided by the AHBM to incorporate inputs related to the vadose zone.

#### Hydraulic conductivity

The hydraulic conductivity (*K*) of an aquifer represents the measure of the rate at which water, and consequently pollutants, can flow through it. As geological conditions can vary significantly from one location to another, the values of *K* also exhibit substantial heterogeneity. Estimating hydraulic conductivity usually involves various methods, such as aquifer tests, pumping tests, laboratory experiments, or field measurements. These approaches provide valuable information for understanding the flow characteristics of water and pollutants within the aquifer (Eq. (6)).

6 $$K= \frac{T}{B}$$

where

*K*: refers to hydraulic conductivity (m/s),
*T*: refers to transmissivity (m^2^/s), and.
*B*: refers to the thickness of the aquifer (m).

Hydraulic conductivity (*K*) is a crucial parameter for predicting the response of an aquifer to recharge and pumping activities (Thapa et al. 2018). High *K*-values indicate a greater flow rate of water, making aquifers more susceptible to pollution. Conversely, low *K*-values result in reduced water flow and make aquifers relatively less vulnerable to contamination. This study obtained the distribution map of hydraulic conductivity from the AHBM, providing valuable information for assessing the vulnerability of the aquifer to pollutants.

### Land use

The vulnerability of an aquifer to pollution is greatly influenced by the land use pattern. Agricultural lands, in particular, have been found to pose a high vulnerability to pollution due to the extensive use of fertilizers and other agrochemicals (Lahjouj et al. 2022). The global land cover data from the European Space Agency, version 2, 2021 (https://esa-worldcover.org/en, last accessed on 11 May 2022), was used in this study to generate the required land use map. This data source provided valuable information on the spatial distribution of land cover types, enabling an assessment of the potential impact of different land use categories on the vulnerability of the aquifer to pollutants.

## Results and discussion

### Identification of hydrology and field conditions in the study area

**Fig. 4:**
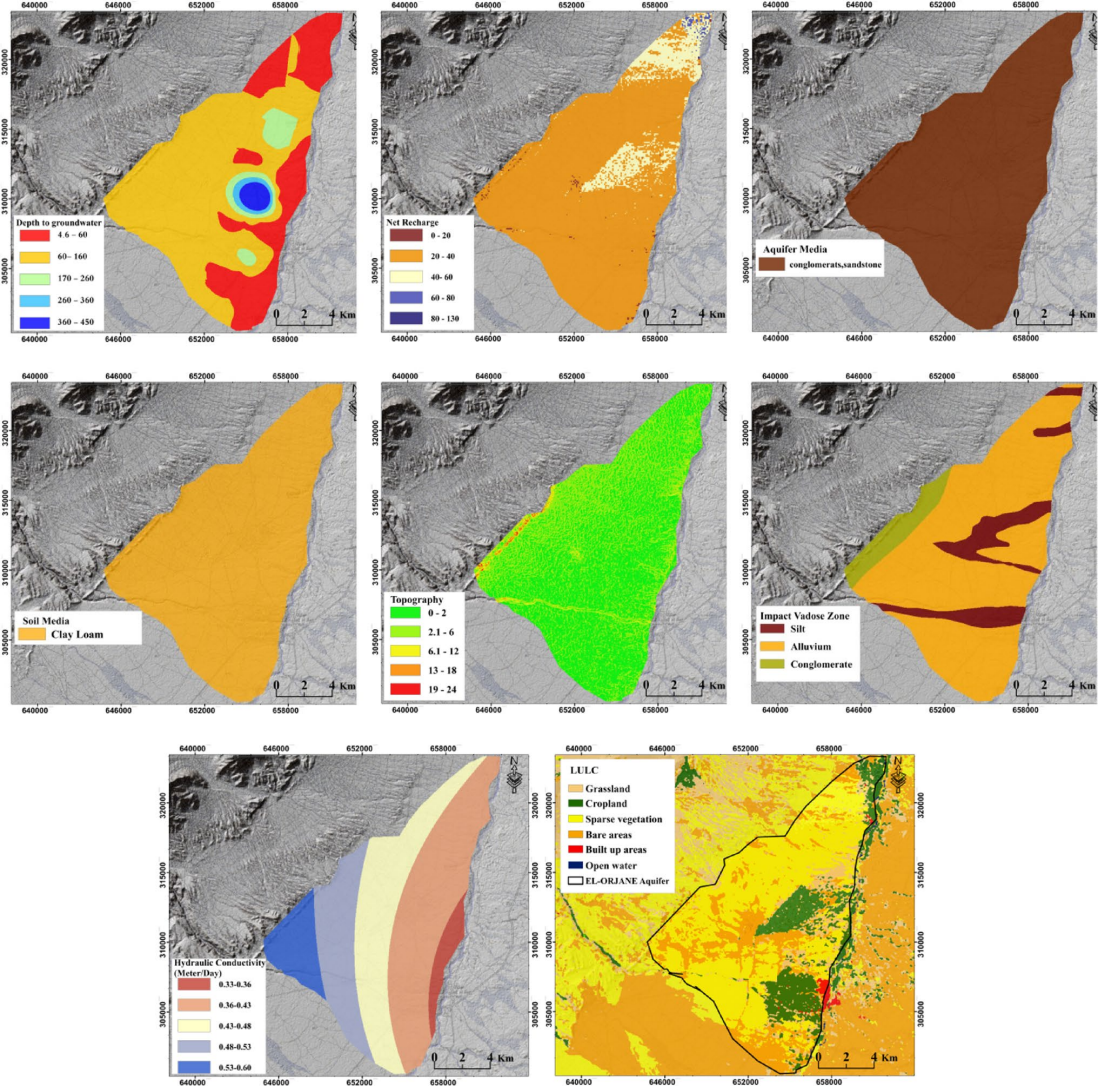
Input maps for generating GVMs using different applied methods

Figure 4 displays the input maps that were prepared for the production of GVMs using different methods. Depths to groundwater in the study area range from 4 to 450 m. Piezometers near the mainstream of the Moulouya Valley recorded the lowest depths, while depths increase as we move away from the main stream. To assign appropriate weights to these groundwater depths, we have classified them into five classes: 4–60 m, 60–160 m, 160–260 m, 260–360 m, and 370–450 m. The weights assigned to these classes are 5, 4, 3, 2, and 1, respectively. A weight of “5” indicates a high vulnerability of the aquifer to pollution, while lower weights indicate decreasing vulnerability. The El Orjane aquifer has a maximum hydraulic conductivity (*K*) value of 0.6 m/day and a minimum value of 0.33 m/day. The annual recharge of this aquifer varies from 0 to 133 mm, with an average value of 33 mm. Most of the study area experiences low recharge rates; however, the zone with agricultural activities exhibits high recharge rates. This high recharge potential increases the potential for groundwater pollution due to the transport of fertilizers from the land surface to the underlying aquifer layers. Lastly, the study area encompasses six types of land use: agricultural area, grassland, bare area, open water, urban settlements, and rural settlements. These land use categories provide valuable information about the surface characteristics and potential sources of pollution that can impact the aquifer.

### Groundwater vulnerability to pollution

#### DRASTIC, Pesticide DRASTIC, and SINTACS models

**Fig. 5:**
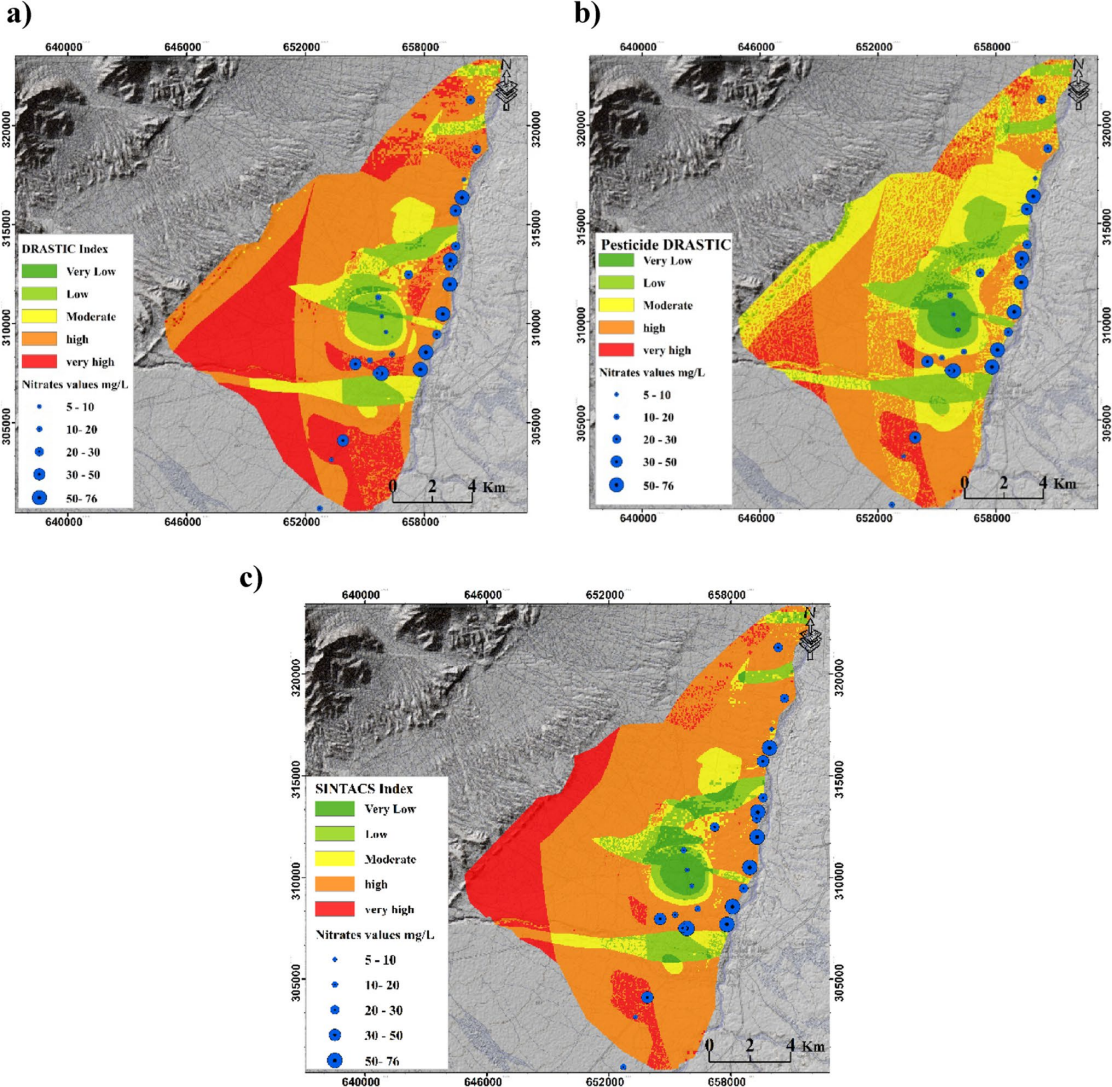
GVMs generated by DRASTIC, Pesticide DRASTIC, and SINTACS models

Figure 5 illustrates the GVMs generated by three models: DRASTIC, Pesticide DRASTIC, and SINTACS. To quantify the differences between the applied models and compare their outputs, groundwater vulnerability was classified into five categories: very low, low, moderate, high, and very high. Based on the results of the DRASTIC and Pesticide DRASTIC models, 0.85% and 3.3% of the study area were classified as very low vulnerability to pollution, 9.44% and 16.34% as low vulnerability, 10.45% and 31.05% as moderate vulnerability, 47.93% and 42.39% as high vulnerability, and 31.31% and 6.89% as very high vulnerability, respectively (see Fig. 5). The results indicate that the modifications made to the parameter weights in the Pesticide DRASTIC model led to an increase in the percentage of areas classified as moderately and highly vulnerable to pollution. This finding is consistent with previous studies, such as Ahmed (2009), who reported higher groundwater vulnerability ratings using the Pesticide DRASTIC model compared to the original DRASTIC model.

The GVMs indicate that 79% of the study area is classified as highly and very vulnerable to pollution (Fig. 5). Moving towards the northwest of the study area, a higher percentage of land is categorized as highly vulnerable. This can be attributed to the presence of lands with high hydraulic conductivity values, primarily composed of conglomerate formations. On the other hand, the SINTACS model reveals that approximately 14.1% of the study area is classified as a low and very low vulnerability, predominantly located in the central region. Table 4 presents the Pearson correlation values (PCV) for the produced GVMs. The PCV for the GVMs generated by the DRASTIC, Pesticide DRASTIC, and SINTACS were 0.42, 0.53, and 0.47, respectively.

**Table 4:** Pearson correlation coefficient for the produced GVMs

Method	Correlation matrix (Pearson)
DRASTIC	0.42
Pesticide DRASTIC	0.53
SINTACS	0.47
DRASTIC-LU	0.28
Pesticide DRASTIC-LU	0.25
SINTACS-LU	0.37
SI	0.43

#### DRASTIC-LU, Pesticide DRASTIC-LU, SINTACS-LU, and SI models 

**Fig. 6:**
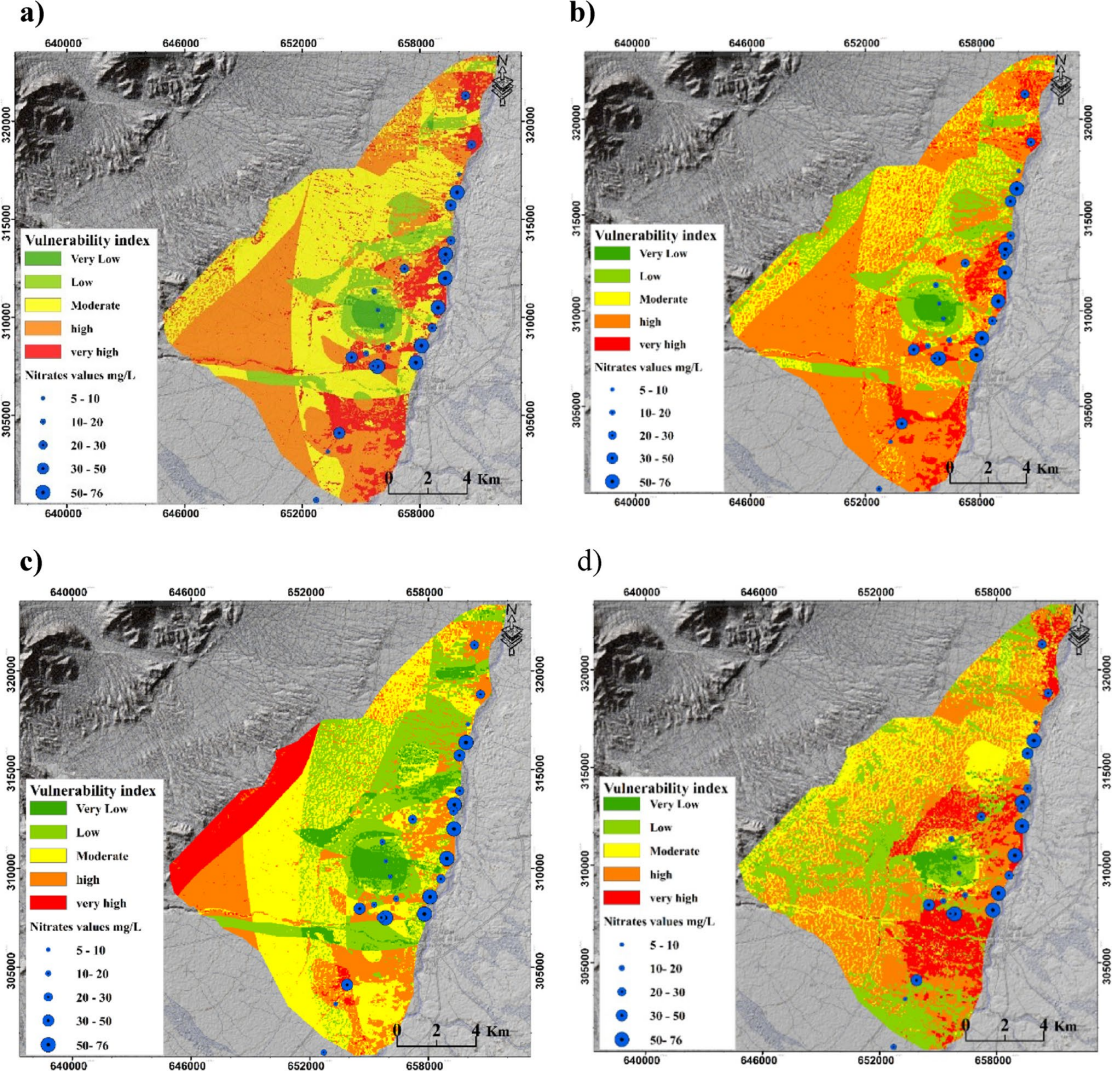
GVMs generated by DRASTIC-LU, Pesticide DRASTIC-LU, SINTACS-LU, and SI methods

Figure 6 depicts the impact of incorporating the land use layer into the DRASTIC, Pesticide DRASTIC, and SINTACS models. The figure also presents the GVMs generated using the SI method, which includes a land use layer as a key component.

The implementation of the land use layer in the DRASTIC, Pesticide DRASTIC, and SINTACS models resulted in notable changes in the classification of zones vulnerable to pollution, as evident from Fig. 6. For instance, the groundwater vulnerability map produced by DRASTIC-LU indicated that only 12.55% of the study area is classified as “highly vulnerable,” whereas the original DRASTIC model predicted it to be 31.31% (Fig. S1). Similar changes were observed in the maps generated by the Pesticide DRASTIC and Pesticide DRASTIC-LU methods, with the percentage of “highly vulnerable” zones increasing from 6.59 to 7.55% after incorporating the land use layer (Fig. S1). Furthermore, the parameter of depth to groundwater was found to exert a significant influence on groundwater VI in all methods. Areas characterized by greater depths to groundwater tended to exhibit lower vulnerability in the resulting maps. In the SI method, land use emerged as the most crucial factor, surpassing even the depth to the groundwater table. This led to the identification of moderate to high vulnerability areas (as shown in Fig. 6) predominantly in agricultural regions, which serve as a significant source of nitrate that poses a threat to aquifer quality. Similar to a study by Armanuos et al. (2019) for the Western Nile Delta aquifer, Egypt, the SI method achieved the highest Pearson value among the four methods with LU, despite not considering soil type, the impact of the vadose zone, and hydraulic conductivity. This underscores the crucial role of agricultural activities and land use patterns in controlling groundwater vulnerability to pollution.

### Sensitivity analysis of the groundwater vulnerability parameters

Table 5 presents the values of parameter weights obtained from the single-parameter sensitivity analysis. The table highlights the most influential parameters identified for each of the different methods used in the study:

- Vadose zone: for DRASTIC, DRASTIC-LU, SINTACS, and SINTACS-LU
- Depth to groundwater: for Pesticide DRASTIC and Pesticide DRASTIC-LU
- Topography: for SI.

**Table 5:** Sensitivity analysis results for parameters influencing groundwater vulnerability assessment

Parameter	Theoretical weight	Theoretical weight (%)	Effective weight			
			Mean	Min	Max	SD
DRASTIC						
*D*	5.00	21.74	21.80	6.41	33.78	3.93
*R*	4.00	17.39	16.35	4.70	23.88	1.81
*A*	3.00	13.04	15.84	12.39	32.43	1.42
*S*	2.00	8.70	6.33	4.95	9.37	0.57
*T*	1.00	4.35	4.83	0.85	7.81	0.75
*I*	5.00	21.74	25.78	16.30	37.03	4.08
*C*	3.00	13.04	9.00	3.03	16.48	2.85
Pesticide DRASTIC						
*D*	5.00	19.23	41.46	15.20	61.59	5.61
*R*	4.00	15.38	25.11	7.40	42.16	3.26
*A*	3.00	11.54	5.76	4.84	7.63	0.36
*S*	5.00	19.23	12.16	10.20	21.76	1.31
*T*	3.00	11.54	5.55	1.07	10.88	0.93
*I*	4.00	15.38	7.67	3.87	14.19	2.10
*C*	2.00	7.69	2.25	0.64	4.43	0.91
DRASTIC-LU						
*D*	5.00	17.86	18.66	4.85	31.25	3.28
*R*	4.00	14.29	13.99	3.63	20.77	1.57
*A*	3.00	10.71	13.54	10.56	19.48	1.03
*S*	2.00	7.14	5.41	4.22	7.79	0.41
*T*	1.00	3.57	4.13	0.75	6.41	0.60
*I*	5.00	17.86	22.12	12.82	33.89	3.88
*C*	3.00	10.71	7.74	2.41	14.70	2.55
LU	5.00	17.86	14.35	4.90	28.08	3.67
Pesticide DRASTIC-LU						
*D*	5.00	16.13	45.93	19.03	61.76	4.67
*R*	4.00	12.90	18.02	4.38	35.83	3.28
*A*	3.00	9.68	6.65	5.16	8.70	0.55
*S*	5.00	16.13	5.43	4.19	11.99	0.83
*T*	3.00	9.68	7.42	1.19	15.81	1.50
*I*	4.00	12.90	8.64	5.59	12.27	1.29
*C*	2.00	6.45	0.78	0.21	1.56	0.32
LU	5.00	16.13	7.09	2.35	13.56	2.07
SINTACS						
*D*	5.00	19.23	19.00	6.00	30.00	3.00
*R*	4.00	15.38	14.00	4.00	21.00	2.00
*A*	3.00	11.54	14.00	12.00	20.00	9.00
*S*	4.00	15.38	12.00	10.00	16.00	1.00
*T*	2.00	7.69	9.00	1.00	13.00	1.00
*I*	5.00	19.23	22.00	14.00	31.00	3.00
C	3.00	11.54	7.00	2.00	15.00	3.00
SINTACS-LU						
*D*	5.00	16.13	17	0.04	0.27	0.03
*R*	4.00	12.90	12	0.03	0.18	0.01
*A*	3.00	9.68	12	0.10	0.16	0.01
*S*	4.00	12.90	10	0.08	0.13	0.01
*T*	2.00	6.45	7	0.02	0.10	0.02
*I*	5.00	16.13	19	0.11	0.25	0.02
*C*	3.00	9.68	7	0.02	0.13	0.03
lu	5.00	16.13	14	0.04	0.25	0.04
SI method						
*D*	0.19	18.60	19.62	4.72	31.83	3.39
*R*	0.21	21.20	21.07	5.24	32.91	2.89
*A*	0.12	12.10	14.13	3.20	22.22	1.99
*T*	0.26	25.90	33.11	26.48	47.57	2.97
lu	0.22	22.20	12.04	0.00	27.75	7.28

**Fig. 7:**
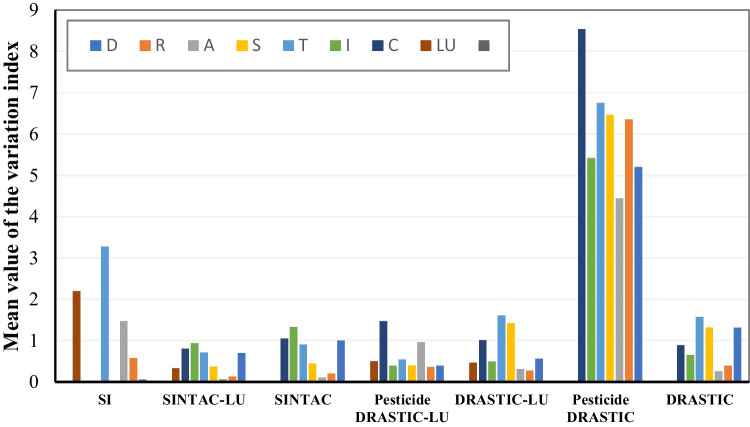
Mean values of the variation index based on the map removal method

Using the map removal method, Fig. 7 illustrates the changes in the mean values of the variation index resulting from the removal of each parameter in the different methods. Table 6 provides a summary of the most sensitive parameters identified through a single-parameter and map removal sensitivity analysis for the applied models. Furthermore, Table S4 presents the variation index for each parameter of the considered methods.

**Table 6:**
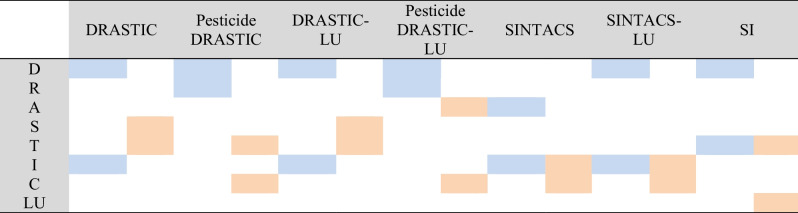
Sensitivity analysis results for the most influential parameters in the applied models (single-parameter and map removal analysis

For Single parameter

For Map removal

### Improvement of the groundwater vulnerability models

#### AHP method

**Fig. 8:**
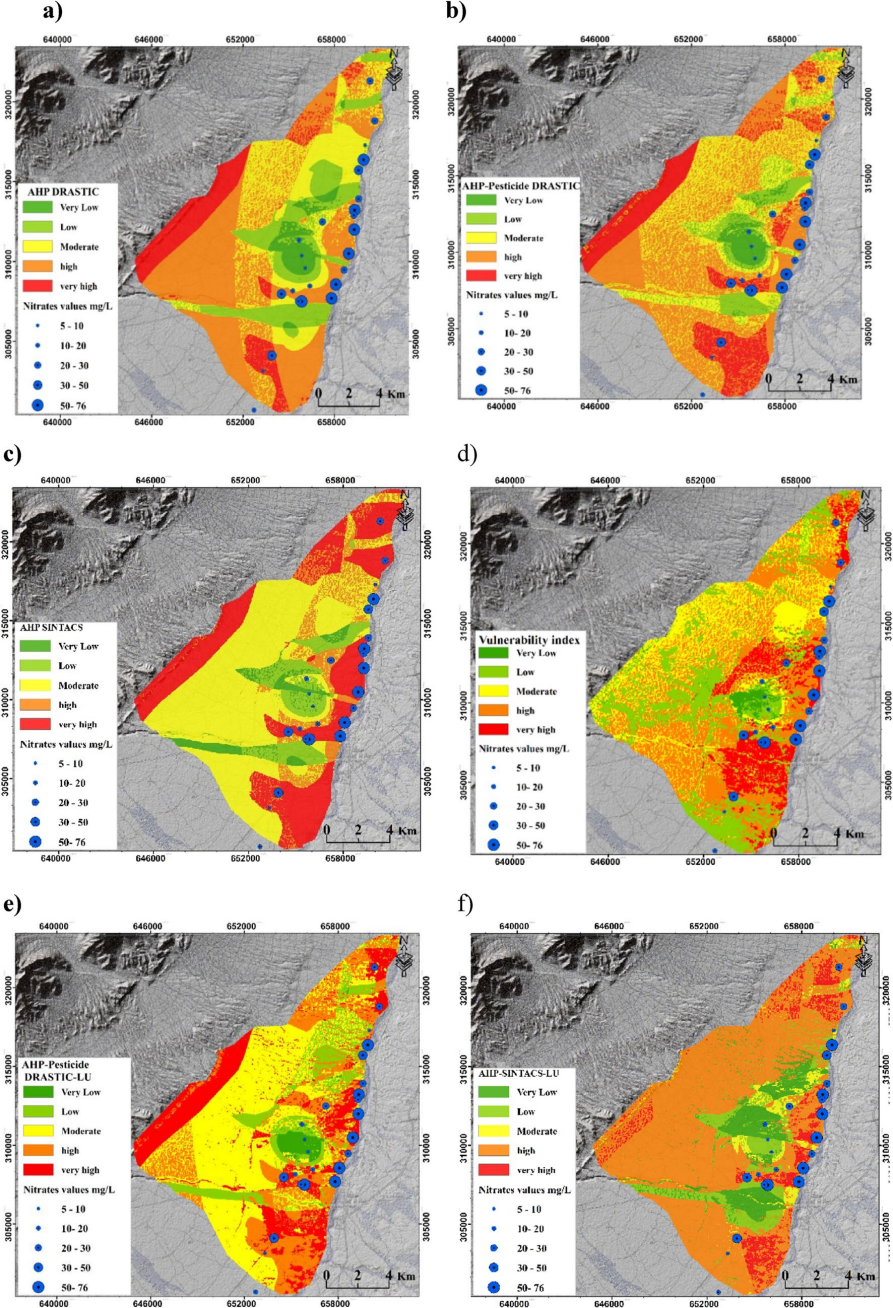
GVMs after improvements using AHP

Based on the results of the sensitivity analysis, the rates and weights of the parameters used to assess groundwater vulnerability were refined (Table S5), leading to improved GVMs with higher values of Pearson correlation. Regarding the present study and to illustrate the improvements resulting from the adoption of the AHP method, Fig. 8 displays the GVMs generated after the application of the AHP method.

As depicted in Fig. 8, significant changes in the vulnerability level of various zones within the study area can be observed. These changes were a direct result of refining the parameter weights through the application of the AHP method. When validating these updated maps using measured nitrate concentrations from 24 locations in the study area, substantial improvements were observed in the PCV (Table 7). For example, the modification of Pesticide DRASTIC-LU through the AHP has increased the PCV by 64%. Similar findings of the efficiency of AHP method in improving groundwater vulnerability assessment to the present results have been reported for the Egirdir Lake basin, Turkey, by Sener and Davraz (2013); for the Kerman Plain, Iran, by Neshat et al. (2014a, b); for the Jharia coalfield, India, by Karan et al. (2018); for the Weibei Plain, China, by Hu et al. (2018), and in central Nile Delta, Egypt, by Metwally et al. (2023). Fig. S2 illustrates the percentage of vulnerable areas for each AHP-generated map.

**Table 7:** Pearson correlation coefficient for the different methods before/after improvements by AHP

Method	Pearson correlation coefficient	
	After AHP	% improvement
DRASTIC	0.51	22%
Pesticide DRASTIC	0.548	3.8%
SINTACS	0.59	25.5%
DRASTIC-LU	0.41	46.4%
Pesticide DRASTIC-LU	0.41	64%
SINTACS-LU	0.59	59.5%

#### Wilcoxon test

**Fig. 9:**
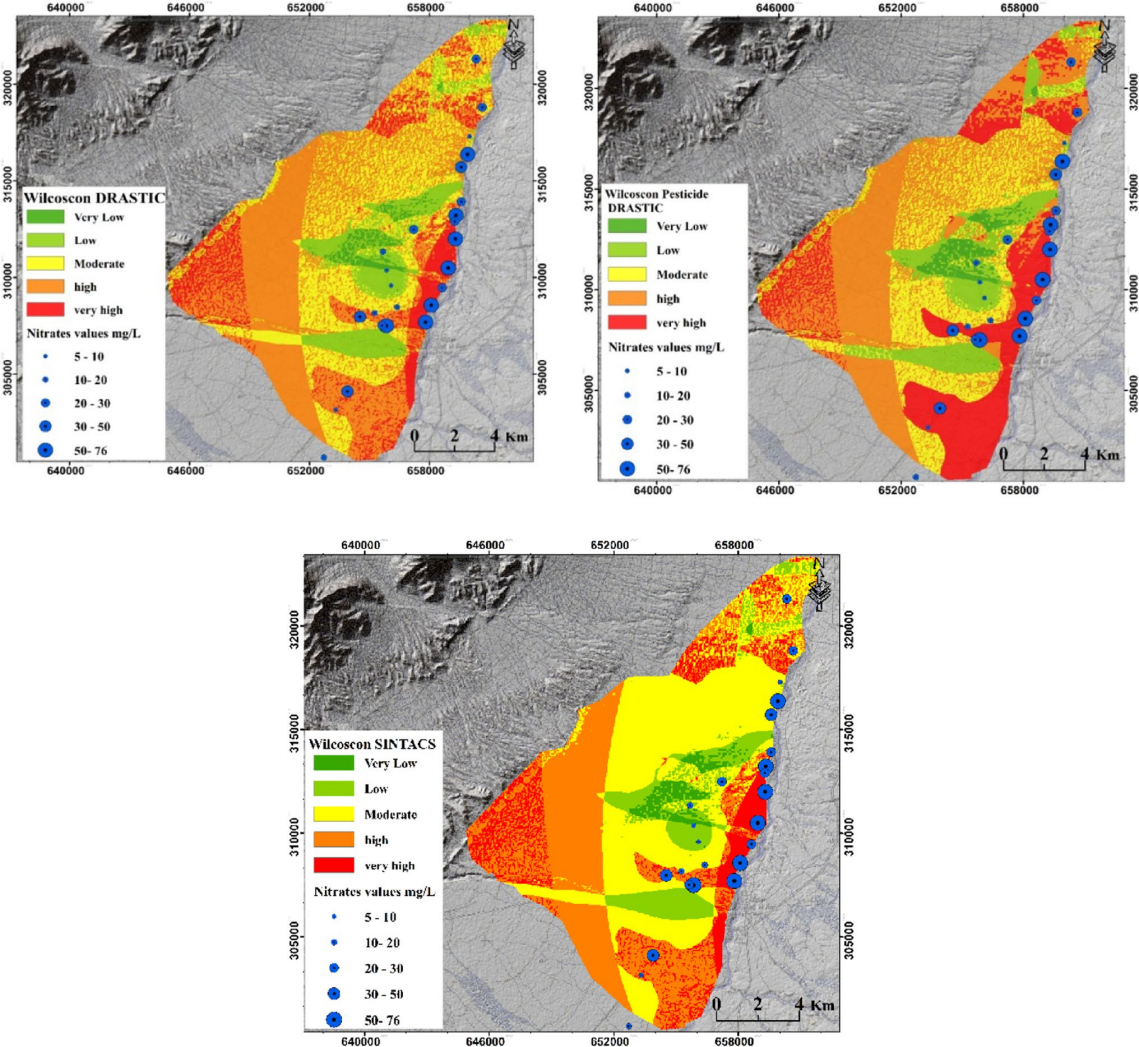
GVMs after improvements using Wilcoxon test for DRASTIC, Pesticide DRASTIC, and SINTACS methods

The modified rates of the parameters, as determined through the Wilcoxon test, successfully increased the correlation between the predicted vulnerability index and the measured nitrate concentration. In the present study, the correlation values for the different methods improved from 0.42 to 0.75, from 0.53 to 0.73, and from 0.47 to 0.74 for DRASTIC, Pesticide DRASTIC, and SINTACS, respectively. Given the highest correlation value (e.g., 0.75) obtained for the Wilcoxon DRASTIC method, the implemented modifications in the parameter rates resulted in significant changes in vulnerability predictions, as shown in Fig. 9. For instance, after applying Wilcoxon to DRASTIC, only approximately 23.94 km^2^ of the study area was classified as high vulnerability zones, compared to the 57.7 km^2^ predicted by DRASTIC before applying the Wilcoxon test. Similarly, for zones of moderate vulnerability, Wilcoxon DRASTIC estimated approximately 37.29 km^2^ as moderately vulnerable zones, instead of the 57.24 km^2^ predicted by DRASTIC before the Wilcoxon test.

**Fig. 10:**
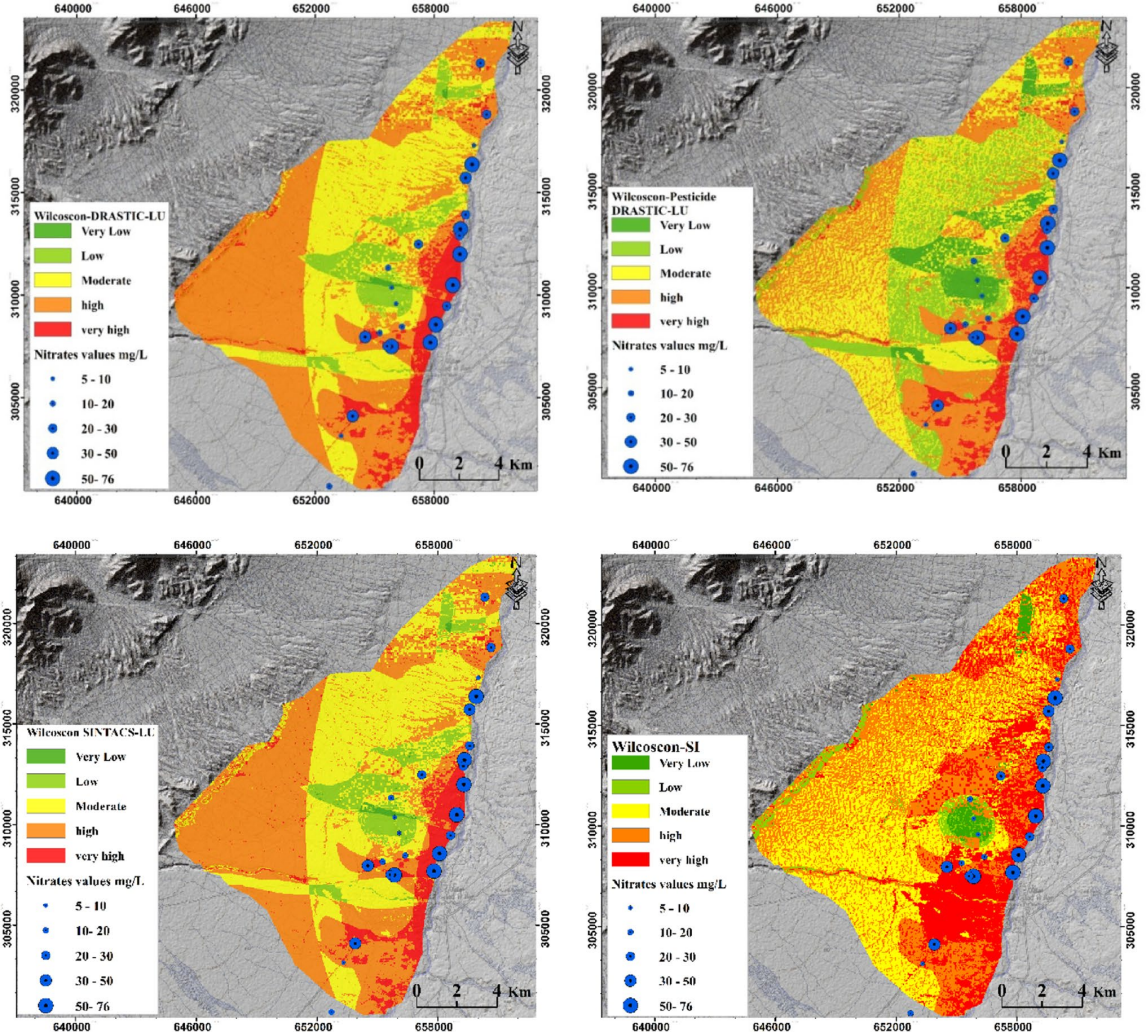
GVMs after improvements using Wilcoxon test for DRASTIC-LU, Pesticide DRASTIC-LU, SINTACS, and SI methods

The Wilcoxon test was also applied to improve the correlation for the DRASTIC-LU, Pesticide DRASTIC-LU, and SINTACS methods. Application of the Wilcoxon test resulted in an increase in correlation for these methods: DRASTIC-LU increased from 38 to 68%, Pesticide DRASTIC-LU increased from 40 to 60%, and SINTACS increased from 38 to 74%. Specifically, for the DRASTIC-LU method, the use of the Wilcoxon test led to an increase in the predicted percentage of highly vulnerable regions in the study area from 33.77 to 55.44% (as shown in Fig. 10 and Fig. S2). Additionally, the percentage of moderately vulnerable regions increased from 19.05% (before Wilcoxon) to 33.84% (after Wilcoxon). Regarding the performance of the Wilcoxon rank-sum nonparametric statistical test in improving groundwater vulnerability assessment, our findings are consistent with those reported by Neshat et al. (2014a, b) for the Kerman agricultural area, Iran, by Balaji et al. (2021) for the Chennai metropolitan area, India, and by Lahjouj et al. (2022) for the Saiss basin, Morocco. These improvements are expected to provide valuable insights for more accurate groundwater vulnerability assessment and contribute to better groundwater and environmental management practices in arid and semi-arid regions.

## Conclusion

The increasing necessity to assess groundwater vulnerability arises from the widespread and continuous development of human activities that adversely impact water resources, particularly groundwater, through leakage. This research paper proposes improvements to GIS-based methods for groundwater vulnerability assessment. The study focuses on the El Orjane aquifer in the Moulouya basin, Morocco, where 24 piezometers were installed to measure nitrate concentrations in groundwater samples. These measurements were used to validate the produced GVMs and evaluate the proposed improvements. The GVMs classify the study area into five vulnerability categories: very low, low, moderate, high, and very high. Pearson’s correlation coefficient was employed to assess the agreement between the GVMs and measured nitrate concentrations. Several improvements were proposed to enhance the accuracy of the assessment. Firstly, a land use layer was incorporated into existing methods (DRASTIC, Pesticide DRASTIC, and SINTACS) to provide valuable information on land use patterns, which significantly influence groundwater vulnerability. Secondly, the AHP and Wilcoxon methods were applied to modify parameter weights in the DRASTIC-LU, Pesticide DRASTIC-LU, SINTACS-LU, and SI methods. These modifications aimed to optimize parameter rates and improve vulnerability assessments. The results revealed significant increases in PCV after implementing the proposed improvements. For example, in the DRASTIC model, the PCV increased from 0.42 to 0.75 after incorporating the land use layer and adjusting parameter rates using the Wilcoxon method. These findings highlight the enhanced accuracy and reliability of groundwater vulnerability assessments achieved through the proposed improvements. However, there are limitations to consider. First, the study focused on a specific aquifer and may not be directly applicable to other regions with different hydrogeological characteristics. The proposed improvements should be evaluated and tailored to specific study areas. Additionally, the validation of vulnerability maps using nitrate concentrations in groundwater samples may not capture the full range of pollutants or represent long-term variations in water quality. Future work should address these limitations and expand the research scope. Conducting comparative studies across multiple aquifers and regions with diverse hydrogeological conditions would provide a more comprehensive understanding of the proposed improvements’ effectiveness. Additionally, integrating other relevant factors, such as pollutant sources and transport mechanisms, into the assessment models could further enhance their accuracy. Furthermore, exploring the applicability of the proposed improvements to other types of contaminants and developing advanced modeling techniques to account for temporal variations in vulnerability would be valuable areas for future research.

In conclusion, with the incorporation of land use information, optimization of parameter weights, and utilization of statistical techniques, the accuracy and reliability of vulnerability assessments can be significantly improved. Such proposed improvements allow better assessment of groundwater vulnerability using the GIS-based methods. However, further research and refinement are needed to ensure the applicability and effectiveness of these improvements across various hydrogeological contexts and pollutant scenarios.

### Supplementary Information

Below is the link to the electronic supplementary material.

Supplementary file1 (DOCX 182 KB)

## Data Availability

The datasets used and/or analyzed during the current study are available from the corresponding author on reasonable request.
