# Supplementary material for: Enhancing groundwater vulnerability assessment for improved environmental management: addressing a critical environmental concern
Source: Environ Sci Pollut Res Int. 2024 Feb 15;31(13):19185–205. doi: 10.1007/s11356-024-32305-1 (PMC10927854; doi:10.1007/s11356-024-32305-1)
Supplement: Supplementary file 1 — Supplementary file1 (DOCX 182 KB) [file 11356_2024_32305_MOESM1_ESM.docx]

**Table S1** Saaty’s 1–9 scale of relative importance (Saaty 1980)

| Scaling | Numerical rating |
| --- | --- |
| Extreme importance | 9 |
| Very to extremely strong importance | 8 |
| Very strong importance | 7 |
| Strong to very strong importance | 6 |
| Strong importance | 5 |
| Moderate to strong importance | 4 |
| Moderate importance | 3 |
| Equal to moderate importance | 2 |
| Equal importance | 1 |

**Table S2** Saaty’s ratio index for different values of n.

| Order | 1 | 2 | 3 | 4 | 5 | 6 | 7 | 8 | 9 | 10 | 11 |
| --- | --- | --- | --- | --- | --- | --- | --- | --- | --- | --- | --- |
| RI | 0.00 | 0.00 | 0.52 | 0.89 | 1.11 | 1.25 | 1.35 | 1.40 | 1.45 | 1.49 | 1.52 |

**Table S3** Map removal sensitivity analysis

|  | Mean | Max | Min | SD |
| --- | --- | --- | --- | --- |
| DRASTIC | | | | |
| D | 1.31 | 0.03 | 3.24 | 0.52 |
| R | 0.39 | 0 | 1.59 | 0.22 |
| A | 0.26 | 0 | 0.97 | 0.14 |
| S | 1.32 | 0.81 | 1.55 | 0.09 |
| T | 1.57 | 1.07 | 2.23 | 0.12 |
| I | 0.65 | 0.13 | 2.4 | 0.52 |
| C | 0.89 | 0 | 1.87 | 0.45 |
| Pesticide DRASTIC | | | | |
| D | 5.2 | 2.61 | 12.03 | 1.22 |
| R | 6.35 | 3.93 | 10.21 | 0.72 |
| A | 4.44 | 2.77 | 6.17 | 0.59 |
| S | 6.46 | 4.7 | 8.95 | 0.62 |
| T | 6.75 | 4.7 | 9.29 | 0.67 |
| I | 5.42 | 2.18 | 8.78 | 0.92 |
| C | 8.54 | 5.76 | 12.34 | 1.03 |
| DRASTIC-LU | | | | |
| D | 0.56 | 0 | 2.67 | 0.44 |
| R | 0.28 | 0 | 2.1 | 0.24 |
| A | 0.31 | 0 | 0.89 | 0.21 |
| S | 1.42 | 0.71 | 1.8 | 0.25 |
| T | 1.61 | 0.97 | 2.25 | 0.26 |
| I | 0.49 | 0 | 1.9 | 0.51 |
| C | 1.009 | 0 | 2.25 | 0.47 |
| lu | 0.46 | 0 | 2.08 | 0.36 |
| DRASTIC-LU | | | | |
| D | 0.56 | 0 | 2.67 | 0.44 |
| R | 0.28 | 0 | 2.1 | 0.24 |
| A | 0.31 | 0 | 0.89 | 0.21 |
| S | 1.42 | 0.71 | 1.8 | 0.25 |
| T | 1.61 | 0.97 | 2.25 | 0.26 |
| I | 0.49 | 0 | 1.9 | 0.51 |
| C | 1.01 | 0 | 2.25 | 0.47 |
| Lu | 0.46 | 0 | 2.08 | 0.36 |
| Pesticide DRASTIC-LU | | | | |
| D | 0.39 | 0 | 2.21 | 0.34 |
| R | 0.36 | 0 | 2.1 | 0.25 |
| A | 0.96 | 0 | 1.69 | 0.29 |
| S | 0.4 | 0 | 0.84 | 0.24 |
| T | 0.54 | 0 | 2.16 | 0.32 |
| I | 0.39 | 0 | 1.88 | 0.35 |
| C | 1.47 | 0.51 | 2.31 | 0.31 |
| Lu | 0.5 | 0 | 1.95 | 0.29 |
| SINTACS | | | | |
| D | 1 | 0.04 | 2.63 | 0.42 |
| R | 0.2 | 0 | 1.67 | 0.18 |
| A | 0.11 | 0 | 0.99 | 0.12 |
| S | 0.45 | 0 | 0.67 | 0.12 |
| T | 0.91 | 0.15 | 2.06 | 0.19 |
| I | 1.33 | 0.04 | 2.82 | 0.49 |
| C | 1.05 | 0 | 1.92 | 0.53 |
| SINTACS-LU | | | | |
| D | 0.7 | 0 | 2.18 | 0.29 |
| R | 0.13 | 0 | 1.3 | 0.13 |
| A | 0.07 | 0 | 0.62 | 0.063 |
| S | 0.37 | 0 | 0.57 | 0.073 |
| T | 0.71 | 0.21 | 1.55 | 0.13 |
| I | 0.93 | 0 | 1.89 | 0.38 |
| C | 0.8 | 0 | 1.46 | 0.41 |
| Lu | 0.33 | 0 | 1.82 | 0.47 |
| SI Method | | | | |
| D | 0.06 | 0.01 | 3.81 | 0.59 |
| R | 0.58 | 0.01 | 3.68 | 0.48 |
| A | 1.47 | 0.11 | 4.19 | 0.49 |
| T | 3.27 | 1.62 | 6.89 | 0.74 |
| Lu | 2.19 | 0.31 | 5 | 1.56 |

**Table S4** Pairwise comparison matrix and normalized weight for AHP methods

| Methods | Item | Depth to groundwater | Recharge | Aquifer media | Soil media | Topography slope | Vadose zone | Hydraulic conductivity | Land use/Land cover | Normalized Weight |
| --- | --- | --- | --- | --- | --- | --- | --- | --- | --- | --- |
| AHP DRASTIC | D | 1.00 | 3.00 | 5.00 | 6.00 | 7.00 | 2.00 | 4.00 |  | 0.34 |
|  | R |  | 1.00 | 3.00 | 4.00 | 5.00 | 0.50 | 2.00 |  | 0.16 |
|  | A |  |  | 1.00 | 2.00 | 3.00 | 0.25 | 0.50 |  | 0.07 |
|  | S |  |  |  | 1.00 | 2.00 | 0.20 | 0.33 |  | 0.05 |
|  | T |  |  |  |  | 1.00 | 0.17 | 0.25 |  | 0.03 |
|  | I |  |  |  |  |  | 1.00 | 0.33 |  | 0.19 |
|  | C |  |  |  |  |  |  | 1.00 |  | 0.16 |
| AHP DRASTIC PESTICIDE | D | 1.00 | 2.00 | 3.00 | 1.00 | 3.00 | 2.00 | 5.00 |  | 0.25 |
|  | R |  | 1.00 | 2.00 | 0.50 | 2.00 | 1.00 | 4.00 |  | 0.15 |
|  | A |  |  | 1.00 | 0.25 | 1.00 | 0.50 | 3.00 |  | 0.09 |
|  | S |  |  |  | 1.00 | 0.50 | 1.00 | 2.00 |  | 0.19 |
|  | T |  |  |  |  | 1.00 | 0.25 | 1.00 |  | 0.10 |
|  | I |  |  |  |  |  | 1.00 | 2.00 |  | 0.17 |
|  | C |  |  |  |  |  |  | 1.00 |  | 0.06 |
| AHP SINTACS | D | 1.00 | 2.00 | 3.00 | 2.00 | 4.00 | 1.00 | 3.00 |  | 0.25 |
|  | R |  | 1.00 | 2.00 | 1.00 | 3.00 | 0.50 | 2.00 |  | 0.14 |
|  | A |  |  | 1.00 | 0.25 | 2.00 | 0.33 | 1.00 |  | 0.08 |
|  | S |  |  |  | 1.00 | 1.00 | 0.25 | 0.50 |  | 0.12 |
|  | T |  |  |  |  | 1.00 | 0.33 | 1.00 |  | 0.07 |
|  | I |  |  |  |  |  | 1.00 | 2.00 |  | 0.25 |
|  | C |  |  |  |  |  |  | 1.00 |  | 0.10 |
| AHP DRASTIC- LU | D | 1.00 | 3.00 | 5.00 | 6.00 | 7.00 | 2.00 | 4.00 | 1.00 | 0.26 |
|  | R |  | 1.00 | 3.00 | 4.00 | 5.00 | 0.50 | 2.00 | 0.50 | 0.13 |
|  | A |  |  | 1.00 | 2.00 | 3.00 | 0.25 | 0.50 | 0.33 | 0.06 |
|  | S |  |  |  | 1.00 | 2.00 | 0.20 | 0.33 | 0.50 | 0.05 |
|  | T |  |  |  |  | 1.00 | 0.17 | 0.25 | 0.33 | 0.03 |
|  | I |  |  |  |  |  | 1.00 | 0.33 | 0.50 | 0.15 |
|  | C |  |  |  |  |  |  | 1.00 | 0.25 | 0.12 |
|  | LU |  |  |  |  |  |  |  | 1.00 | 0.20 |
| AHP DRASTIC pesticide- LU | D | 1.00 | 2.00 | 3.00 | 1.00 | 3.00 | 2.00 | 5.00 | 1.00 | 0.20 |
|  | R |  | 1.00 | 2.00 | 0.50 | 2.00 | 1.00 | 4.00 | 0.50 | 0.12 |
|  | A |  |  | 1.00 | 0.25 | 1.00 | 0.50 | 3.00 | 0.33 | 0.07 |
|  | S |  |  |  | 1.00 | 0.50 | 1.00 | 2.00 | 0.50 | 0.14 |
|  | T |  |  |  |  | 1.00 | 0.25 | 1.00 | 0.33 | 0.08 |
|  | I |  |  |  |  |  | 1.00 | 2.00 | 0.50 | 0.13 |
|  | C |  |  |  |  |  |  | 1.00 | 0.25 | 0.05 |
|  | LU |  |  |  |  |  |  |  | 1.00 | 0.21 |
| AHP SINTAC- LU | D | 1.00 | 2.00 | 3.00 | 2.00 | 4.00 | 1.00 | 3.00 | 1.00 | 0.20 |
|  | R |  | 1.00 | 2.00 | 1.00 | 3.00 | 0.50 | 2.00 | 0.50 | 0.11 |
|  | A |  |  | 1.00 | 0.25 | 2.00 | 0.33 | 1.00 | 0.33 | 0.06 |
|  | S |  |  |  | 1.00 | 1.00 | 0.50 | 1.00 | 0.50 | 0.11 |
|  | T |  |  |  |  | 1.00 | 0.33 | 0.33 | 0.33 | 0.05 |
|  | I |  |  |  |  |  | 1.00 | 2.00 | 0.50 | 0.17 |
|  | C |  |  |  |  |  |  | 1.00 | 0.33 | 0.08 |
|  | LU |  |  |  |  |  |  |  | 1.00 | 0.21 |


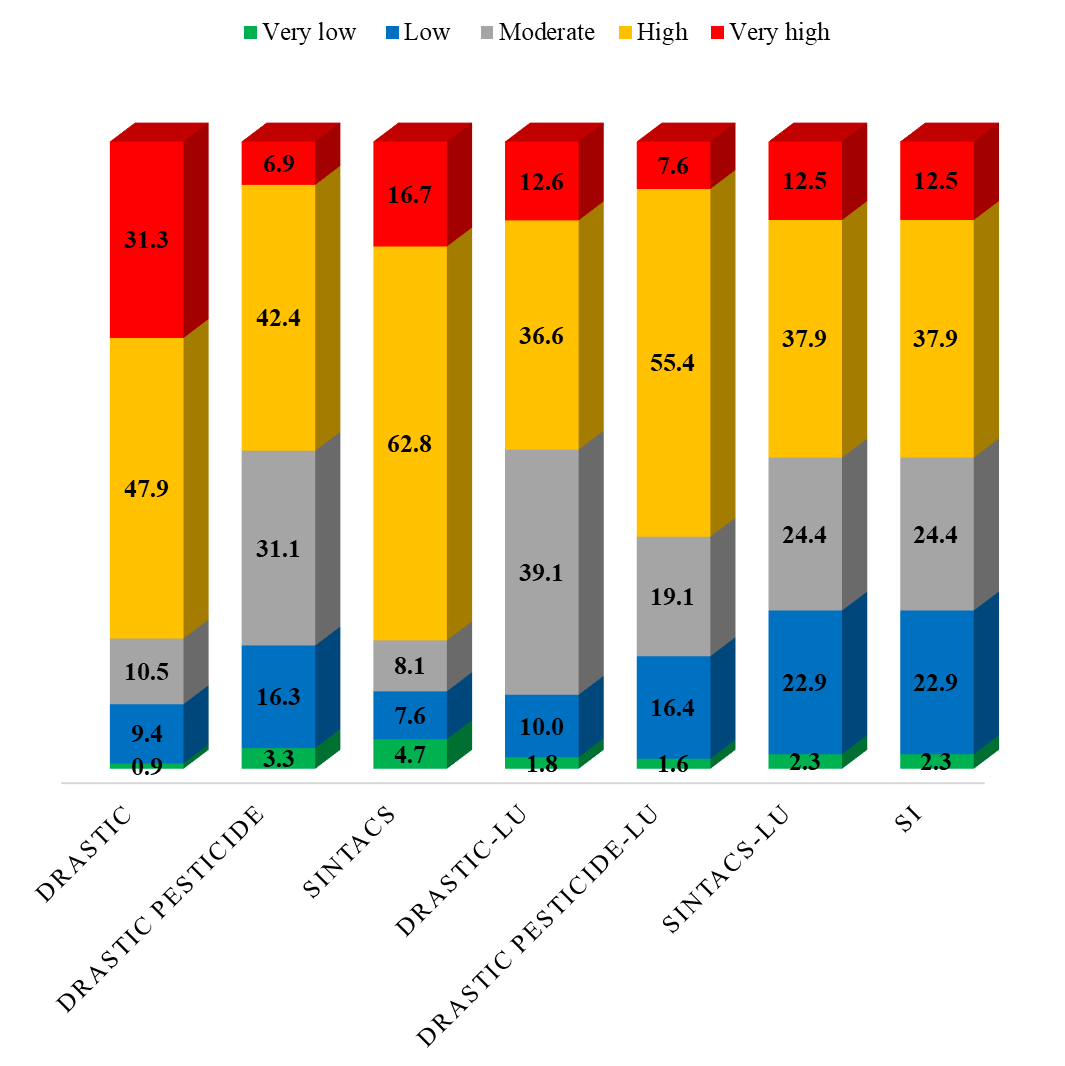


**Fig. S1** Percentage of Vulnerable areas in DRASTIC, DRASTIC Pesticide, SINTACS, DRASTIC-LU, DRASTIC Pesticide-LU, SINTACS-LU, and SI methods.


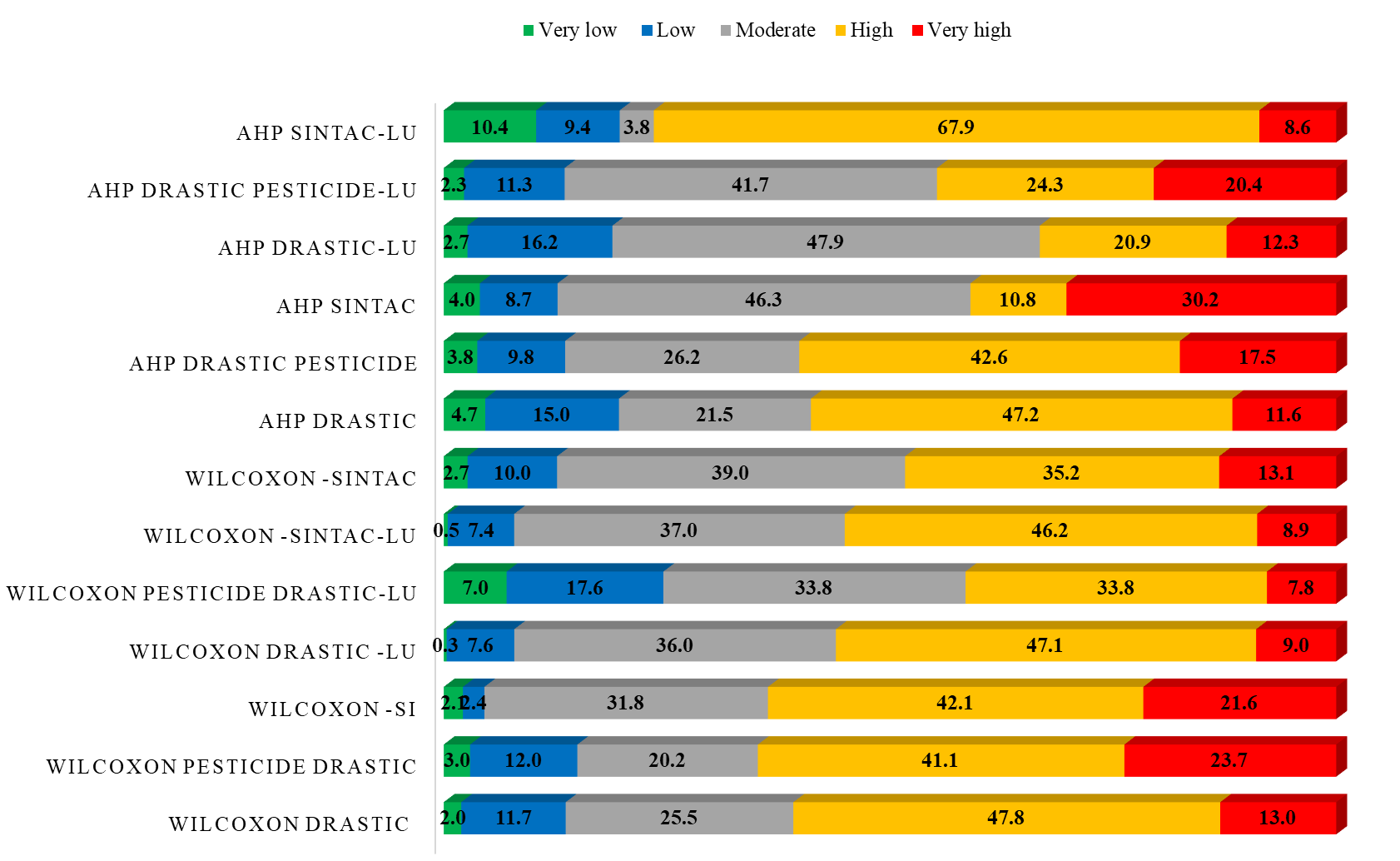


**Fig. S2** Percentage of Vulnerable areas in AHP DRASTIC, AHP DRASTIC PESTICIDE, AHP SINTACS, AHP DRASTIC-LU, AHP DRASTIC pesticide-LU, AHP SINTAC-LU, Wilcoxon DRASTIC, Wilcoxon Pesticide Drastic, Wilcoxon -SINTAC, Wilcoxon-SI, Wilcoxon DRASTIC -LU, Wilcoxon Pesticide Drastic-LU, and Wilcoxon-SINTAC-LU methods.
